# Detection of SARS-CoV-2 by rapid antigen tests on saliva in hospitalized patients with COVID-19

**DOI:** 10.1080/20008686.2021.1993535

**Published:** 2021-10-29

**Authors:** Yang De Marinis, Anne-Katrine Pesola, Anna Söderlund Strand, Astrid Norman, Gustav Pernow, Markus Aldén, Runtao Yang, Magnus Rasmussen

**Affiliations:** a Department of Clinical Sciences Malmö, Lund University, Malmö, Sweden; b School of Control Science and Engineering, Shandong University, Jinan, China; c Department of Endocrinology and Metabolism, Division of Life Sciences of Medicine, University of Science and Technology of China, Hefei, China; d Clinical Microbiology, Skåne University Hospital, Lund, Sweden; e Division of Infection Medicine, Department of Clinical Sciences Lund, Lund University, Lund, Sweden; f School of Mechanical, Electrical and Information Engineering, Shandong University, Weihai, China

**Keywords:** COVID-19, SARS-CoV-2, antigen test, saliva

## Abstract

**Background:**

The COVID-19 pandemic presents great challenges on transmission prevention, and rapid diagnosis is essential to reduce the disease spread. Various diagnostic methods are available to identify an ongoing infection by nasopharyngeal (NPH) swab sampling. However, the procedure requires handling by health care professionals, and therefore limits the application in household and community settings.

**Objectives:**

In this study, we aimed to determine if the detection of SARS-CoV-2 can be performed alternatively on saliva specimens by rapid antigen test.

**Study Design:**

Saliva and NPH specimens were collected from 44 patients with confirmed COVID-19. To assess the diagnostic accuracy of point-of-care SARS-CoV-2 rapid antigen test on saliva specimens, we compared the performance of four test products.

**Results:**

RT-qPCR was performed and NPH and saliva sampling had similar Ct values, which associated with disease duration. All four antigen tests showed similar trend in detecting SARS-CoV-2 in saliva, but with variation in the ability to detect positive cases. The rapid antigen test with the best performance could detect up to 67% of the positive cases with Ct values lower than 25, and disease duration shorter than 10 days.

**Conclusion:**

Our study therefore supports saliva testing as an alternative diagnostic procedure to NPH testing, and that rapid antigen test on saliva provides a potential complement to PCR test to meet increasing screening demand.

## Introduction

Coronavirus disease (COVID-19) caused by SARS-CoV-2 has led to over 241 million reported infection cases and 4.9 million deaths worldwide [1]. COVID-19 has had a tremendous impact on public health, economics, and social activities; and affects many aspects of daily life. One of the most important prevention and control measurements is rigorous testing and tracking of SARS-CoV-2 in the general population.

Different diagnostic methods have been developed by targeting the genetic material or major structural proteins of SARS-CoV-2. RT-PCR detects the presence of viral RNA in a specimen, whereas antigen test detects the presence of viral proteins. Both methods are applied to confirm ongoing infection and are often performed on nasopharyngeal (NPH) swab samples [2–4].

The rapid antigen tests for the detection of SARS-CoV-2 have been applied in various clinical settings and population screenings. This method is based on a rapid chromatographic immunoassay and designed for the qualitative detection of specific antigens of SARS-CoV-2. The rapid antigen test is not restricted to centralized testing and can be used as a fast point-of-care diagnostic method. Nevertheless, most commercially available antigen tests are designed to be performed on NPH swab samples [4,5]. This procedure causes discomfort for the patients and requires health-care professionals, and therefore limits its application on self-testing in the general population [6].

Previous studies have shown that SARS-CoV-2 can be detected in saliva of asymptomatic carriers, outpatients, as well as hospitalized patients [7–10]. A recent investigation compared viral load by reverse transcriptase digital polymerase chain reaction (RT-dPCR) in saliva, sputum, NPH swab, oropharyngeal swab, anal swab, and feces; and showed that NPH had the highest positive rate (93%) among the six types of specimens, followed by saliva detection (86%) [9].

In this study, we explored the possibility to detect SARS-CoV-2 in saliva specimens as an alternative to NPH samples. We also investigated the application of rapid antigen tests on saliva specimens with respect to disease duration and RT-qPCR Ct values.

## Methods

### Study cohort participants

A total of 44 hospitalized patients with COVID-19 diagnosed at Skåne University Hospital (Lund, Sweden) provided written informed consent to participate in this study. COVID-19 had been confirmed with a positive RT-PCR test on NPH swab specimen at hospital admission. After consent to participate, approximately 1 ml of saliva in a sterile plastic tube and a new NPH-swab in 1 ml of sterile 0.9% sodium chloride solution from the patients were obtained and stored at −80°C until analysis in-batch. Disease duration in days and severity according to need for supportive care were identified through the medical record system.

### Detection of SARS-CoV-2 by RT-qPCR

Among 44 NPH samples collected, detection of SARS-CoV-2 in saliva in 41 of the NPH swab specimens was performed by RT-qPCR, using the MagNA pure 96 automated platform (Roche Life Science). This was followed by PCR-analysis of the envelope gene using a 7500 Fast Real-time PCR system (Applied Biosystems) as previously described [11] with modifications. The PCR cycling program was 48°C for 10 min, 95°C for 10 min and 45 cycles of 95°C for 15 s and 55°C for 45 s. PCR-analyses were performed using the Path-ID Multiplex one-step kit (Thermo-Fisher Scientific) and expressed as Cycle of threshold (Ct). One NPH swab specimen was analysed using a non-commercial extraction technique followed by PCR analysis targeting the envelope gene using a QuantStudio 7 instrument (Applied Biosystems). Two NPH swab specimens were analyzed using the BioFire FilmArray Respiratory 2.1 Panel (BioMerieux).

### Detection of SARS-CoV-2 by rapid antigen tests

Saliva samples from the cohort participants were thawed to room temperature prior to testing. Four SARS-CoV-2 rapid antigen tests were used in this study: Flowflex (Flowflex SARS-CoV-2 Rapid Antigen Test, ZetaGene-Hughes Healthcare, Sweden/UK), Panbio (Panbio COVID-19 Ag Rapid Test, Abbott, UK), Joinstar (Joinstar COVID-19 Antigen Rapid Test (Colloidal gold), China), PCL (PCL COVID-19 Ag Antigen Gold Saliva Lateral Flow Test, Korea). Saliva samples were collected by the swabs provided in respective kits, except for the PCL where saliva and buffer were mixed 1:1 by pipetting. Testing was performed following instructions from respective manufacturer.

### Ethics

The study was performed in accordance with the Declaration of Helsinki. Patients in the study cohort included gave informed consent to sample collection and study participation. The study was approved by the national ethics review board (2020–01747, and 2020–05361 for amendment).

### Statistical analysis

Wilcoxon signed-rank test was performed to compare RT-PCR values from NPH and saliva of the patients. Pearson’s correlation coefficient analysis was performed to measure the degree of correlation between each two random variables among saliva-PCR-Ct values, NPH-PCR-Ct values, and symptom duration.

### Data and resource availability

Data presented in this manuscript are available upon reasonable request to the corresponding author, with the exception of sensitivity data according to GDPR current regulations.

## Results

### Detection of SARS-CoV-2 measured by RT-qPCR in saliva and nasopharyngeal swab specimens and their association with COVID-19 duration

In total 44 patients participated in this study and patient characteristics are given in Table 1. In 40 patients where both NPH and saliva RT-PCR Ct values were available, we compared detection of SARS-CoV-2 in NPH and saliva swab specimens in each patient (Figure 1(a)). The Ct-value was significantly lower in NPH than saliva (*p* < 0.01). In patients where Ct-values for both types of specimen were available and above the threshold for detection (Ct < 40), the Ct-values in saliva correlated with those of NPH (n = 32, Pearson’s correlation coefficient *r* = 0.64, *p* < 0.001, Figure 1(b)). Ct-values of RT-PCR analysis in both NPH and saliva PCR correlated with COVID-19 duration (*r* = 0.58, *p* < 0.001; and *r* = 0.53, *p* < 0.001, respectively, Figure 1(c-d)). These findings suggest that saliva specimens could be a feasible alternative for SARS-CoV-2 detection, especially early in the disease course.

**Table 1.** Baseline characteristics of the cohort participants

Total number	44
Age (years)	N
<60	19
≥60, <75	13
≥75	12
Sex	N (%)
Male	22 (50%)
Female	22 (50%)
COVID-19 duration (Days)	N
Days ≤5	7
5< Days ≤10	22
Days >10	15
COVID-19 severity	N
Home quarantine	2
Hospitalized w/o oxygen therapy	19
Hospitalized with oxygen therapy	19
High flow nasal cannula	4
Intensive care	0

**Figure 1.**
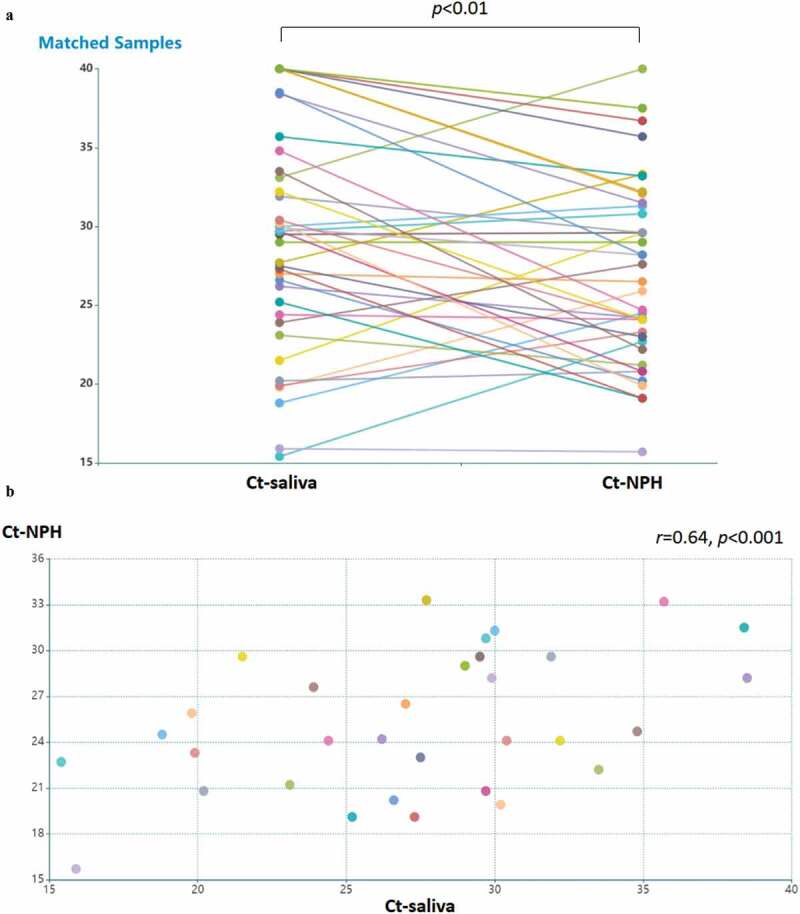
Detection of SARS-CoV-2 in saliva specimens and nasopharyngeal (NPH) swab specimens. samples were obtained from 44 patients with COVID-19. (a) detection of SARS-CoV-2 is presented by RT-PCR Ct values in patients with available NPH and saliva samples (n = 40). the lines indicate samples from the same patient. Ct values in NPH are significantly lower than those in saliva (*p* < 0.01 by Wilcoxon signed-ranked test). Pearson’s correlation coefficient was performed on RT-PCR Ct values above detection threshold (Ct<40) in (b) NPH and saliva samples Ct values (n = 32; *r* = 0.64, *p* < 0.001); (c) NPH Ct values and symptom duration (n = 40; *r* = 0.58, *p* < 0.001); (d) saliva Ct values and symptom duration (n = 34; *r* = 0.54, *p* < 0.001)

**Figure 1.**
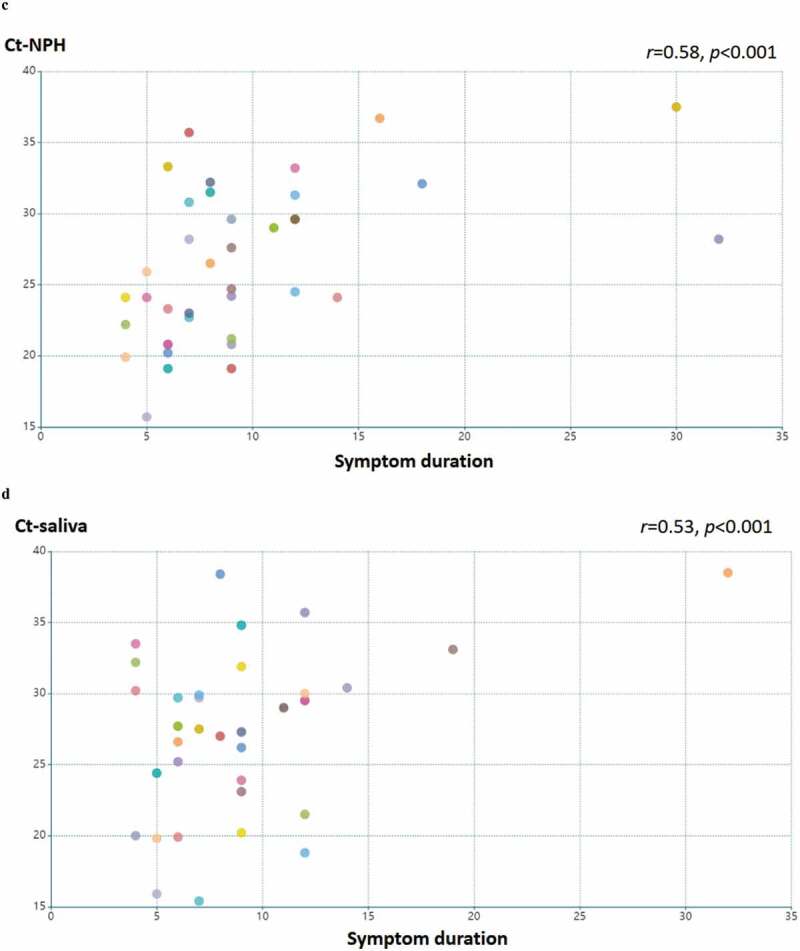
(Continued)

### SARS-CoV-2 rapid antigen testing in saliva specimens with respect to PCR Ct values and COVID-19 duration

**Figure 2.**
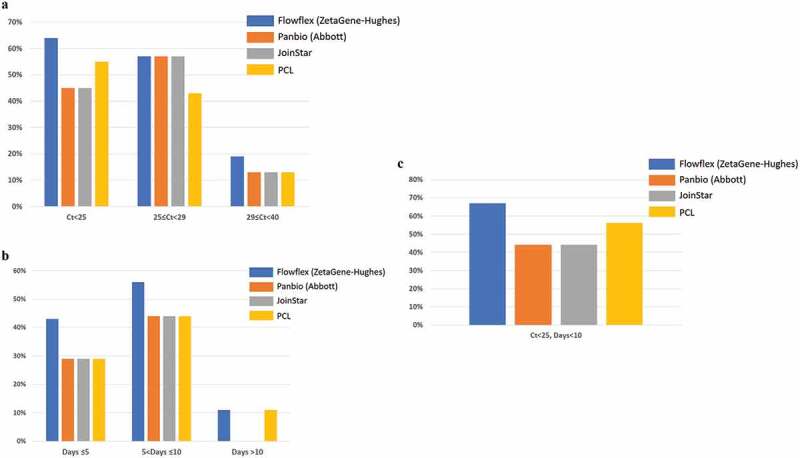
Positive detection rate of SARS-CoV-2 in saliva samples by four SARS-CoV-2 rapid antigen tests: Flowflex (Flowflex SARS-CoV-2 rapid antigen test, ZetaGene-Hughes healthcare, Sweden/UK; blue bars), Panbio (Panbio COVID-19 ag rapid test, Abbott, UK; Orange bars), Joinstar (Joinstar COVID-19 antigen rapid test, China; grey bars), PCL (PCL COVID-19 ag antigen gold saliva lateral flow test, Korea; yellow bars). Saliva specimens were collected from patients with confirmed COVID-19 (n = 44), and 34 samples were above the threshold of detection by RT-PCR (Ct<40). Patients were grouped according to (a) PCR Ct values: Ct<25; 25≤ Ct<29; 29≤ Ct<40; (b) days after COVID-19 symptoms onset: Days≤5; 5< Days≤10; Days≥10; (c) Ct<25, Days<10

We then performed rapid antigen test using saliva specimens from the cohort participants. For saliva samples with RT-PCR Ct values lower than 25, Flowflex tests (ZetaGene-Hughes) were able to detected 64% (7 of 11) of positive cases, followed by PCL (55%, 6 of 11), Panbio (Abbott, 45%, 5 of 11) and Joinstar (45%, 5 of 11) (Table 2, Figure 2(a)). In the Ct-range between 25 and 29 of the saliva samples (n = 7), the positive detection was 57% by Flowflex, Panbio and Joinstar; and 43% by PCL (Table 2, Figure 2(a)). In the Ct range higher than 29 (n = 16), the positive detection rate was further reduced to 19% (Flowflex), 9% (Abbott), 13% (Joinstar) and 13% (PCL) (Table 2, Figure 2(a)).

**Table 2.** Positive detection of SARS-CoV-2 on saliva samples by rapid antigen tests

Saliva-PCR	Flowflex (ZetaGene-Hughes)	Panbio (Abbott)	Joinstar	PCL
Ct<25	64% (7/11)	45% (5/11)	45% (5/11)	55% (6/11)
25≤ Ct<29	57% (4/7)	57% (4/7)	57% (4/7)	43% (3/7)
29≤ Ct<40	19% (3/16)	13% (2/16)	13% (2/16)	13% (2/16)
COVID-19 duration				
Days ≤5	43% (3/7)	29% (2/7)	29% (2/7)	29% (2/7)
5< Days ≤10	56% (10/18)	44% (8/18)	44% (8/18)	44% (8/18)
Days >10	11% (1/9)	0% (0/9)	0% (0/9)	11% (1/9)
Saliva-PCR and COVID-19 duration				
Ct<25, Days<10	67% (6/9)	44% (4/9)	44% (4/9)	56% (5/9)

COVID-19 duration of 5 days or less (n = 7) corresponded to positive detection rate of 43% by Flowflex; and 29% by Panbio, Joinstar and PCL (Table 2 and Figure 2(b)). COVID-19 duration of more than 5 days but less than 10 days (n = 18) corresponded to higher positive detection rate: 56% by Flowflex; 44% by Panbio, Joinstar and PCL (Table 2 and Figure 2(b)). In patients with COVID-19 duration longer than 10 days (n = 9), Flowflex and PCL detected 11% of the positive cases, while Panbio and Joinstar detected none (Table 2 and Figure 2(b)).

Rapid antigen test offered the highest sensitivity when both saliva PCR values and disease duration were taken into consideration. In patients with saliva Ct values lower than 25 and disease duration shorter than 10 days (n = 9), 67% of the positive cases were detected by Flowflex, 44% by Panbio and Joinstar, and 56% by PCL (Table 2 and Figure 2(c)). Overall, Flowflex had the highest sensitivity in detecting viral antigen in saliva specimens from COVID-19 patients.

## Discussion

We show that saliva specimens have similar levels of SARS-CoV-2 as compared to NPH swab specimens in patients admitted to hospital with COVID-19. RT-qPCR Ct values of both saliva and NPH correlated with disease duration. We also show that rapid antigen test using saliva samples can detect SARS-CoV-2 in a subset of patients, mainly those with higher SARS-CoV-2 levels and shorter duration of symptoms.

Detection of SARS-CoV-2 by RT-PCR is still considered ‘gold standard’, while antigen tests for rapid diagnosis have gained an increasingly important role. Antigen tests detect viral proteins and suffer from a lower sensitivity than RT-PCR but are still very useful in certain clinical situations. For rule-in infection purposes, antigen tests have the advantage of being very fast. In the Swedish health care system, antigen tests are extensively used for screening of persons without symptoms of COVID-19 who have been exposed to infection or who have an increased risk of infection. However, a negative antigen test in a person with symptoms compatible with COVID-19 is not enough to exclude infection. It is worth to mention that this study does not address the issue of antigen test specificity, but rather with focus on sensitivity.

The relatively lower rate of positive case detection of rapid antigen tests in this study may be a consequence of several factors. Firstly, the cohort investigated in this study is not ideal to evaluate the usefulness of saliva antigen tests since the tests are intended to be used on persons without known COVID-19. Our results indicate, however, that saliva antigen tests are more likely to be positive early during the disease process and possibly the relatively low positive detection rate demonstrated in this study would be increased if subjects were recruited earlier during COVID-19. Similar studies using different patient cohorts to address these questions are ongoing. Second, with the exception of the antigen test from PCL, the tests used were validated for NPH sampling (Panbio and Joinstar) or nasal sampling (Flowflex). Therefore, the performance of the tests in this validation likely differs from the performance of the test when used according to the protocol. Thirdly, it has been shown that positive results of PCR may not necessarily reflect the presence of viable virus [12]. It is possible that PCR Ct values may not correspond to antigen test results, especially in longer disease duration where residues of viral genetic material from dead virus may be detected by PCR but not by antigen test. Finally, antigen test in this study was performed on saliva samples stored at −80°C and thawed to room temperature. It is recommended by the manufacturers that the testing is preferably performed on freshly collected samples, and therefore the freeze and thaw procedure may have an impact on testing sensitivity.

Currently, the antigen test is often performed on samples taken from NPH, and the sampling procedure requires handling by healthcare professionals. To increase the possibility to test a larger number of subjects, it would be a great advantage if antigen tests could be performed on saliva samples. This offers the possibility of self-testing, as well as much less discomfort in the sampling procedure. Based on our observation of lower, yet similar, detection of SARS-CoV-2 in saliva and NPH samples, we find saliva a promising candidate substrate for rapid SARS-CoV-2 antigen test. It is also worth mentioning that the performance of the rapid tests varies among products and that tests with higher positive detection rates should be preferred.

In conclusion, our study provides support for the potential application of saliva testing as an alternative diagnostic procedure to NPH swab COVID-19 testing. Collection of saliva samples can be easily performed by patients themselves, and therefore reduces direct interaction between patients and health care professionals. This allows for expansion of screening for SARS-CoV-2 in larger populations.
